# Supplementary material for: Cross-biobank generalizability and accuracy of electronic health record-based predictors compared to polygenic scores
Source: Nat Genet. 2025 Aug 27;57(9):2136–45. doi: 10.1038/s41588-025-02298-9 (PMC12425822; doi:10.1038/s41588-025-02298-9)
Supplement: Supplementary file 1 — Supplementary methods and results. [file 41588_2025_2298_MOESM1_ESM.pdf]

nature genetics

Supplementary information https://doi.org/10.1038/s41588-025-02298-9

Cross-biobank generalizability and accuracy of electronic health record-based predictors compared to polygenic scores

In the format provided by the authors and unedited

Supplementary Tables......1
Supplementary Methods......3
Registry data......3
FinnGen......3
UK Biobank......3
EstB......4
Additional Predictors......4
Number of phecodes......4
CCI......4
Supplementary Results......5
Extended Data Figure 1......5
Extended Data Figure 2......5
Extended Data Figure 3......5
Extended Data Figure 4......6
Extended Data Figure 5......6
Extended Data Figure 6......6
Extended Data Figure 7......6
Extended Data Figure 8......7
Extended Data Figure 9......8
Extended Data Figure 10......8
Supplementary Figure 1......9
References......11

Supplementary Tables

Supplementary Table 1. Number of individuals aged 32-70 in each study.

Supplementary Table 2. Number of cases and controls for each disease and study.

SupplementarySupplement Table 3. Excluded phecodes from the predictors for each disease.

Supplementary Table 4. HRs from the Cox-proportional hazards models. In the p-value column, blue shading highlights p-values<0.05, green shading p-values after Bonferroni correction for one study (N=13 tests) <0.05/13, and yellow shading p-values after Bonferroni correction for all three studies (N=3*13) <0.05/(13*3).

Supplementary Table 5. P-values of beta differences. In the p-value column, blue shading highlights p-values<0.05, green shading p-values after Bonferroni correction for one study (N=13 tests) <0.05/13, and yellow shading p-values after Bonferroni correction for all three studies (N=3*13) <0.05/(13*3).

Supplementary Table 6. C-index of the Cox-proportional hazards models.

Supplementary Table 7. P-values of the c-index increases. In the p-value columns, blue shading highlights p-values<0.05, green shading p-values after Bonferroni correction for one

study (N=13 tests) <0.05/13, and yellow shading p-values after Bonferroni correction for all three studies (N=3*13) <0.05/(13*3).

Supplementary Table 8. Area under the precision-recall curves (AUPRCs) for the full 8-year prediction period.

Supplementary Table 9. Correlation (Pearsons' r) the PheRS and PGS, the PheRS and unique number of phecodes recorded during the observation period (1/1/1999 - 31/12/2008), and the PheRS and CCI. In the p-value columns, blue shading highlights p-values<0.05, green shading p-values after Bonferroni correction for one study (N=13 tests) <0.05/13, and yellow shading p-values after Bonferroni correction for all three studies (N=3*13) <0.05/(13*3).

Supplementary Table 10. Correlation (Pearsons' r) of the internally (FinnGen)- and externally-trained PheRS predictions.

Supplementary Table 11. Phecode prevalences in each study during the observation period (1/1/1999 - 31/12/2008) NAs indicate that less than 5 individuals have a diagnosis.

Supplementary Table 12. PheRS coefficients in each study. Phecodes with a prevalence <1% in a study have NAs. For the rank, the coefficients are sorted in descending order, with the largest coefficient in a model thus having a rank of 1.

Supplementary Table 13. Median PheRS coefficients across the three biobank studies. Only those phecodes (phenotypes) that were included in at least 7/13 PheRS models in a biobank study are included.

Supplementary Table 14. Harmonized definitions of flagship diseases using ICD-10 and ICD-9 codes.

Supplementary Table 15. Genome-wide association study summary statistics used to compute PGS and sample overlap with each biobank.

Supplementary Table 16. Mapping of ICD-10 codes to phecodes.

Supplementary Table 17. Mapping of study specific education codes to ISCED-2011

SupplementarySupplement Table 18. Nagelkerke's pseudo-R2s. Measurement of goodness of fit compared to a null model, used to approximate the proportion of variance explained by the model.

Supplementary Table 19. FinnGen authors.

Supplementary Table 20. Estonian Biobank Research Team.

Supplementary Methods
Registry data
FinnGen
The FinnGen data was the same as was used by Jermy *et al.*[1], the following copied from the Supplementary Material.
Phenotype data within FinnGen is constructed from the collection of nationwide electronic health registers. This gives a comprehensive coverage of almost all of a patient's interactions with the health service including hospitalizations, medications, procedures and deaths. The 18 different registers used by the project are listed below in order of their follow-up times:

- Finnish Cancer Registry - From 1953
- Register of Congenital Malformations - From 1963
- Reimbursement - From 1964
- Population Register - From 1964
- Finnish Registry for Kidney Diseases - From 1964
- Causes of Death - From 1969
- Care Register for Health Care Inpatient Visits, HILMO - From 1969
- Socio-economic data - From 1970
- The Finnish Registry of Visual Impairment - From 1983
- Medical Birth Register - From 1987
- Finnish National Infectious Disease Register - From 1989
- Cervical Cancer Screening - From 1991
- Breast Cancer Screening - From 1992
- Drug Purchases - From 1995
- The Care Register for Social Welfare - From 1995
- Care Register for Health Care, specialist outpatient visits, HILMO - From 1998
- Register of Primary Health Care Visits, Avohilmo - From 2011
- The Finnish Vaccination Register - From 2011

Note: while primary health care visits are included within FinnGen, by default these cases are excluded from the endpoints. As such, we only consider secondary care data for our disease endpoints.
UK Biobank
All data is based on hospital records. We considered all diagnoses marked as primary, secondary and tertiary and took the earliest date of diagnoses from any of the columns.
Data columns used:

- HESIN the hospital inpatient admission with the main table (field 1063) containing information on all hospital inpatient admissions and the diagnosis table (field 1067) covering all diagnosis codes recorded in an inpatient admission. The date was based on the start of the hospital stay. (See also https://biobank.ndph.ox.ac.uk/ukb/refer.cgi?id=593)
- Summary ICD-10 main diagnoses (field 41202 with date 41262)
- Summary ICD-9 main diagnoses (field 41203 with date 41263)
- Summary ICD-10 secondary diagnoses (field 41204 with date 41280)
- Summary ICD-9 secondary diagnoses (field 41205 with date 41281)
- Cause of death (field 40001 for main ICD-10 and 40002 for secondary ICD-10 with dates from 40000)

Additional variables were extracted from:

- Date of birth: based on month of birth (field 52), year of birth (field 34) and then the 15th of each month.
- PCs: Genetic principal components from (field 22009)
- End of followup: Based on information from date of death (field 40000)
- Education: Qualifications (field 6138) mapped to ISCED-11 with this mapping: Supplementary Table 16

All elements were constructed using information from 0 - initial assessment visit (2006-2010) at which participants were recruited and consent given, if 0 not available then 1 - first repeat assessment visit (2012-13) and otherwise 2 - imaging visit (2014+) if neither 0 or 1 contained information.

For the code and further documentation see: GitHub - UKB phenotyping.

EstB

The EstB data was the same as was used by Jermy *et al.*[1], the following copied from the Supplementary Material.

Phenotype data within EstBB is put together from the collection of electronic health registers, including from two largest hospitals in Estonia. We include both primary and secondary care data as well as self-reported diagnoses. Estonia has a solidary health insurance system and national public health insurance covers ~94% of the population (https://eurohealthobservatory.who.int/countries/estonia). Causes of death and Cancer registry record all cases despite the health insurance status in Estonia. Following registries were included in phenotype definition process:

- Causes of Death Registry- diagnoses from 2003 until 2020
- National Cancer Registry- diagnoses from 1955 until 2017
- Estonian Health Insurance Fund - From 2001 until 2020
- The North Estonia Medical Centre from 1993 until 2017
- Tartu University hospital from 2006 until 2017
- E-Health system from 1998 to 2020

Self-reported diagnoses' dates ranged from 1920 - 2018.

Additional Predictors

Number of phecodes

The unique number of phecodes is a simple count of the number of phecodes for which an individual had at least one recorded diagnosis during the observation period.

CCI

The Charlson comorbidity index (CCI[2]) assigns fixed weights to a set of comorbid conditions which increase an individual's relative risk of dying. The original version had 19 comorbidity categories, and later Deyo et al.[3] combined the malignancies leukaemia and lymphomas into a single category reducing it to 17 categories. The weights were based on the estimators of a Cox-PH model, adjusted for other diseases, illness severity, and the hospital admission reason. The final CCI score of an individual is the sum of all of their comorbidity weights. For our analyses, we used the adaptations of the CCI to ICD-9 and ICD-10 codes using the *comorbidity* package in R. The code can be found at https://github.com/dsgelab/ICCI.

Supplementary Results

Extended Data Figure 1.

In FinnGen, we found c-index improvements for 10/13 diseases when adding the PheRS to a baseline model with age and sex (asthma, epilepsy, knee OA, T2D, MDD, hip OA, CHD, AF, gout, and breast cancer); in the UKB for 13/13 diseases, and in the EstB for 7/13 (MDD, asthma, knee OA, hip OA, T2D, gout, AF; **Extended Data** Figure 1a-1). When comparing to an extended baseline model including, additionally a simple count of the unique number of recorded phecodes during the observation period, we find that the PheRS improves over this extended baseline model for 9/13, 11/13 and 4/13 diseases in FinnGen, UKB and EstB, respectively. With the greatest reduction in c-index improvements compared to the baseline with only age and sex in the EstB (**Extended Data** Figure 1a-2). When considering the c-index of the model with only PheRS as predictors, the results are very similar to the HRs of the PheRS (**Figure 2a**). We find the highest predictive discrimination for gout (c-index=0.66; 95% CI: 0.64-0.69), lung cancer (c-index=0.61; 95% CI: 0.58-0.64), and MDD (c-index=0.62; 95% CI: 0.59-0.65). It is interesting to note that lung cancer is the only disease we consider where even with high HRs and good predictive discrimination, the PheRS only significantly improve prediction over age and sex in the UKB study (**Extended Data Figure 1a**).

Extended Data Figure 2.

Overall, integrating the CCI only lead to minor improvements in the models' discriminative ability, with marginal significant improvements for asthma, epilepsy, CHD, and gout in FinnGen (4/13); MDD, epilepsy, T2D, CHD, lung cancer, AF, in the UKB (7/13); and asthma, MDD, and Gout in the EstB (3/13; **Extended Data Figure 2a**). Only three of these results were statistically significant after multiple hypothesis testing corrections within each study (N=13). The PheRS and CCI were moderately correlated (average Pearsons' r=0.19, range 0.00-0.46, Supplementary Table 9, **Supplementary Figure 1d**). As the CCI is a weighted sum of selected existing conditions (see Supplementary Methods), it is to be expected that PheRS, being essentially a weighted sum of all existing conditions but where weights are learned from the data, outperforms CCI in predicting disease outcomes. Similarly, integrating education level only lead to minor improvements in the model's discriminative ability, with minor significant improvements in FinnGen (4/13) and the UKB (5/13) (**Extended Data Figure 2b**).

Extended Data Figure 3.

We find that the HRs of the models with PheRS are significantly larger than those of the number of diagnoses for 13/13 diseases in FinnGen, 13/13 in the UKB, and 6/13 in the EstB (**Extended Data** Figure 3a). While the two predictors are strongly correlated (**Extended Data** Figure 1c), the magnitude of the PheRS HRs reduces only slightly for 1/13 diseases in FinnGen, 0/13 in the UKB, and 5/13 in the EstB. We can intuit that the PheRS captures the number of phecodes and other more complex patterns of the data in a way that the contributions are not easily detangled. This is supported by the fact that the HRs of the number of unique phecodes attenuates when adding the PheRS to the models (Supplementary Table 4). We have noticed similar patterns

between PheRS and age, which is why we regress out the effect of age and sex to give a more realistic picture of what the PheRS captures beyond these important predictors.

Extended Data Figure 4.

As previously reported[4], we find significantly higher HRs for the PGS in younger individuals (32-51) for 5/13 (prostate, breast cancer, T2D, AF, and hip OA). For the other 8/13 we find no significant difference to the older age group (52-70).

Extended Data Figure 5

Extended Data Figure 5 shows the c-index improvements of the externally-trained PheRS over age and sex in FinnGen and the UKB, compared to the internally-trained PheRS. With the UKB-trained models we see significant improvements for 8/13 diseases (nominal p-value <0.05; asthma, epilepsy, T2D, hip OA, knee OA, MDD, CHD, and Gout) over baseline and for hip OA the improvements were not significantly different to those achieved by the FinnGen-trained PheRS. With the EstB-trained models we see significant improvements for 5/13 diseases (p-value <0.05; asthma, knee OA, T2D, MDD, and gout) over baseline in FinnGen, with also only the hip OA improvements not significantly different to the FinnGen-trained PheRS. Notably, the EstB-trained models tested in the UKB showed improvements that were not statistically different to the UKB-trained PheRS for 5/13 (p-value <0.05; T2D, hip OA, knee OA, AF, gout, and prostate cancer). For 10/13 the models significantly increased the c-index over the baseline model (nominal p-value<0.05).

Extended Data Figure 6.

In this sensitivity analysis, we train additional consensus PheRS models across the studies with only phecodes common (prevalence of at least 1%) to all three studies. When considering internal testing, we see a significant decrease in performances for 7/13, 13/13 and 8/13 models in FinnGen, UKB and EstB, respectively, with only one minor improvement for breast cancer in the UKB. However, interestingly, when considering external testing, we find a stronger association for 8/13 diseases in FinnGen with the EstB-trained models and only for 2/13 diseases a weaker association. This is likely explained by the similarity of the EstB and FinnGen data, with the main difference being the wider availability of primary care data in EstB compared to FinnGen. For the other models, the changes are comparatively minor. Performance in the external test set decreases significantly for epilepsy and gout when testing the UKB-trained models in Finngen, for asthma and gout when testing the EstB-trained models in Finngen and for knee OA, epilepsy and lung cancer when testing the EstB-trained models in UKB.

Extended Data Figure 7.

This Figure shows the results of a leave-one-out analysis performed in FinnGen. We sequentially removed each of the top 20 predictors (ranked by absolute weight in the original elastic net model) and evaluated the performance of the resulting ridge regression models. Ridge regression was used due to the computational complexity of fitting multiple elastic net models with hyperparameter tuning and we have previously observed that, in particular in

FinnGen, there is little performance difference between ridge and elastic-net models (**Extended Data Figure 7a)**. The analysis shows that removing some predictors, such as "chronic airway obstruction" (in the lung cancer model) and "hypertension" (in the gout model), led to significant reductions in AUC, indicating these features carry unique predictive information. In contrast, the removal of most other top predictors resulted in only minimal changes in model performance (**Extended Data Figure 7b)**. This observation reflects the highly correlated nature of phecodes and the complex interplay of risk factors, which are only partially captured in EHR data. Overall, the LOO analysis highlights that no single predictor dominates the model and that the models distribute weights across correlated features effectively.

Extended Data Figure 8.

For gout the PheRS models in all three studies capture the risk factors hypertension (code 401, FinnGen rank 3, UKB rank 3, EstB rank 3), overweight (code 278, FinnGen rank 7, UKB rank 5, EstB rank 4), and diabetes (code 250, FinnGen rank 11, UKB rank 12, EstB rank 5). Notably, in FinnGen the phecode *stillbirth* (code 634) is the fourth most important predictor after *hypertension* (code 401, rank 88 in EstB and rank 134 in the UKB) and *urticaria* (code 947, rank 7) is an important predictor in the EstB PheRS model with neither stillbirth nor urticaria having an immediately clear connection to gout. In the UKB model, *renal failure* (code 585, rank 4) is the most important predictor after age, sex, and hypertension, and this phecode was not included in the other studies (<1% prevalence in 32-70 year olds in FinnGen and EstB).

The PheRS models for asthma captured various phecodes related to respiratory infections or inflammations with *other symptoms of respiratory system* (code 512) the most important predictor in FinnGen before age and sex (UKB rank 3, EstB rank 17). This phecode captures diagnoses such as *cough* (ICD-10 R05) and *dyspnoea* (ICD-10 R06.0). In the EstB, on the other hand, the most important predictor was *acute bronchitis and bronchiolitis* (code 483, FinnGen rank 10) which could be better captured due to the availability of diagnoses from primary health care. In the UKB, the phecode *other diseases of respiratory system, not elsewhere classified* (code 519, UKB rank 5) further illustrates the difference in coding between the studies, as this phecode had a prevalence of less than 1% in the other two studies and includes diagnoses such as *unspecified acute lower respiratory infection* (ICD-10 J22) which overlaps with conditions captured by the phecode for *acute bronchitis and bronchiolitis*. Further, the models captured other phecodes related to allergies, such as *allergic rhinitis* (code 476, FinnGen rank 45, EstB rank 3), *urticaria* (code 947, EstB rank 16), *atopic/contact dermatitis due to other or unspecified* (code 939, FinnGen rank 4, EstB rank 11), and *chronic sinusitis* (code 475, FinnGen rank 4, EstB rank 11). Similarly to the MDD PheRS models, the asthma models in each study seem to capture the underlying risk factors using multiple phecodes, overall enabling better transferability between the studies. In the UKB, however, many of the top predictors in the other models have a prevalence of less than 1%. Instead, the model includes phecodes such as *overweight* (code 278, UKB rank 4, EstB rank 14, FinnGen rank 47) and *poisoning by analgesics, antipyretics, and antirheumatics* (code 965, UKB rank 6) as top predictors, while high BMI has been previously reported as a risk factor[5] the interpretation of *poisoning by analgesics, antipyretics, and antirheumatics* in relation to asthma is not immediately clear.

For knee OA many of the most important predictors were consistent important predictors across the three studies. These include phecodes for *other peripheral nerve disorders* (code 351, rank FinnGen rank 4, UKB rank 6, EstB rank 7), *peripheral enthesopathies and allied syndromes* (code 726, rank 5 in all models), *other disorders of synovium, tendon, and bursa* (code 727, FinnGen rank 8, UKB rank 5, and EstB rank 44), *spondylosis and allied disorders* (code 721, FinnGen rank 22, UKB rank 13, EstB rank 9), *injury (NOS)* (code 1009, FinnGen rank 3, UKB rank 15, EstB rank 10), *overweight* (code 279, FinnGen rank 9, UKB and EstB rank 2)*, hypertension* (code 401, FinnGen rank 26, UKB rank 7, EstB rank 3), and *varicose veins* (code 454, FinnGen rank 7, UKB rank 20, EstB rank 8). In FinnGen and the UKB, the phecodes *pain in limb* (code 773, FinnGen rank 12, UKB rank 11) and *pain in joint* (code 745, FinnGen rank 6, UKB rank 3, EstB rank 47) were among the top predictors. While *osteochondropathies* (code 732) had rank 11 in the EstB models and was not prevalent in the other two studies.

Extended Data Figure 9.

Both PGS and PheRS add information on top of the other predictor, age, and sex. Due to sample overlap with the GWASs, PGS could only be calculated for 4 diseases in UKB (see Methods for details). The HRs of the PGS per 1-SD were significantly larger than those of the PheRS for 5/13 diseases in the meta-analysis (nominal p-value<0.05; T2D, AF, colorectal cancer, breast cancer, prostate cancer; **Extended Data Figure 9a-1**). When adding PheRS to a model with PGS, age, and sex, the HRs of the PGS do not change (**Extended Data 9a-2**). In all three studies, the PheRS and PGS were correlated for most diseases. However the correlation was small with an average Pearsons' *r* of 0.022 (**Figure 5B, Extended Data Figure 9b,** Supplementary Table 9). The overall variance explained by the scores was low, with the highest Nagelkerke pseudo-R^2 for prostate cancer around 0.02 but largely consistent across the three studies (Supplementary Table 18). When interpreting these results, it is important to keep in mind that we predicted the disease onset in a very specific 8-year window and excluded all individuals with an earlier onset of disease. Additionally, the pseudo-R^2 does not have a defined confidence interval and is not directly comparable to the coefficient of determination R^2 of a linear regression. With the exception of T2D, the correlation tended to be lower between the two scores the more predictive the PGS were. This indicates that the low correlation between PheRS and PGS is not just due to the low predictiveness of the two scores. Further, adding PGS to a model with PheRS, age, and sex led to significant improvements for 11/13 in FinnGen, 3/4 in the UKB, and 4/13 in the EstB. In the meta-analysis only the improvements for breast cancer and AF were significant (nominal p-value<0.05; **Extended Data Figure 9c**). This can be explained by the difference in c-index for the baseline models with age and sex, making a meta-analysis of the delta c-indices difficult.

Extended Data Figure 10.

In the meta-analysis PheRS had significantly (p<0.05) larger HRs per 1-SD for 3/13 diseases (epilepsy, MDD, lung cancer) and PGS significantly larger HRs for 6/13 diseases (colorectal cancer, breast cancer, prostate cancer, T2D, AF), for the other diseases there was no significant difference between the two predictors. Overall, we we found the largest association of the PGS

for prostate cancer (meta-analyzed HR: 1.80, 95% CI: 1.75-1.85), T2D (meta-analyzed HR: 1.70, 95% CI: 1.64-1.77), and gout (meta-analyzed HR: 1.1.64, 95% CI: 1.59-1.69).

Supplementary Figure 1

We found that being in the top 10% of the CCI distribution, after regressing out age and sex, led to the largest HRs for lung cancer (meta-analyzed HR=2.09, 95% CI: 1.60-2.58), gout (meta-analyzed HR=2.07, 95% CI: 1.90-2.24), and epilepsy (meta-analyzed HR=1.83, 95% CI: 1.75-1.90) when compared to the rest of the population (**Supplementary Figure 1B-1**, Supplementary Table 3). The top 10% corresponds largely to individuals with a CCI>=2 and a few younger ones with a CCI of 1. A lower education level (ISCED-11<5) was only significantly associated with 5 out of the 13 diseases in all three studies, with the highest relative risk for lung cancer (meta-analyzed HR=1.81, 95% CI: 1.39-2.23), epilepsy (meta-analyzed HR=1.36, 95% CI: 1.18-1.55), T2D (meta-analyzed HR=1.34, 95% CI: 1.18-1.51; **Supplementary Figure 1B,** Supplementary Table 4) in all three studies. The CCI and education level combined, lead to significant improvements in c-index over age and sex for 3/13 diseases (gout, epilepsy, and MDD) in all three studies (**Supplementary Figure 3A-3**). One notable improvement is the increase in c-index for MDD in the UKB (delta c-index 0.038, p=7.71e-26). The PheRS, however, still capture additional information on top of these two predictors as seen in **Supplementary Figures 1A**.

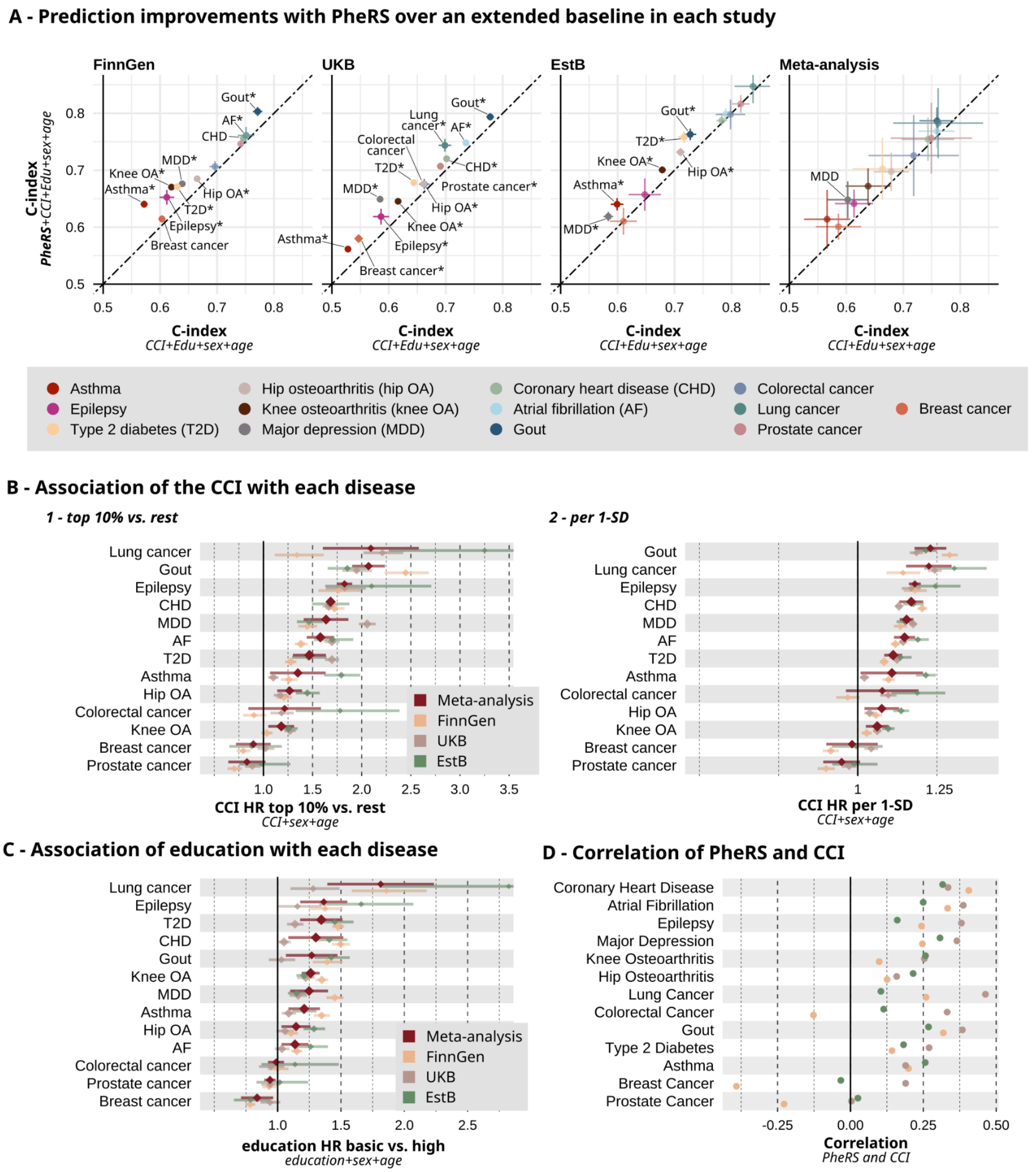

Panel A: Increase in prediction accuracy when adding the PheRS to an extended baseline with age, sex, CCI, and education in all three studies. Association of the CCI with each disease in a model without (x-axis) and with the PheRS (y-axis). Diseases with significant increases in c-index are labelled (p-values < 0.05; one-tailed p-values based on the z-scores of the c-index increases) and those passing multiple hypothesis testing (p-values < 0.05/13) are marked with *. **Panel B**: The HRs of the CCI for the top 10% of individuals with the highest score compared to the rest 90%. Age and sex were regressed-out from the CCI. The top 10% corresponds largely to individuals with a CCI>=2 and a few younger ones with a CCI of 1. **Panel C:** This shows the

increase in relative risk (HR and 95% CI) with 1-SD increase of the CCI. **Panel d**: Association of lower education with each disease. Here we show the increase in relative risk (HR and 95% CI) for individuals with lower education (ISCED-11<5) compared to those with high achieved education level (ISCED-11>=5). **Panel E:** Correlation of the PheRS and CCI after regressing-out the effect of age and sex.

References

1. Jermy, B. *et al.* A unified framework for estimating country-specific cumulative incidence for 18 diseases stratified by polygenic risk. *Nat. Commun.* **15**, 5007 (2024).
2. Charlson, M. E., Pompei, P., Ales, K. L. & MacKenzie, C. R. A new method of classifying prognostic comorbidity in longitudinal studies: development and validation. *J. Chronic Dis.* **40**, 373–383 (1987).
3. Deyo, R. A., Cherkin, D. C. & Ciol, M. A. Adapting a clinical comorbidity index for use with ICD-9-CM administrative databases. *J. Clin. Epidemiol.* **45**, 613–619 (1992).
4. Mars, N. *et al.* Polygenic and clinical risk scores and their impact on age at onset and prediction of cardiometabolic diseases and common cancers. *Nat. Med.* **26**, 549–557 (2020).
5. Liang, H. *et al.* Association of genetic risk and lifestyle with incident adult-onset asthma in the UK Biobank cohort. *ERJ Open Res.* **9**, 00499–02022 (2023).
